# Effervescent Granules Prepared Using* Eucommia ulmoides* Oliv. and Moso Bamboo Leaves: Hypoglycemic Activity in HepG2 Cells

**DOI:** 10.1155/2016/6362094

**Published:** 2016-08-28

**Authors:** Xiang-Zhou Li, Sheng Zhang

**Affiliations:** ^1^College of Materials Science and Engineering, Central South University of Forestry and Technology, Changsha, Hunan 410004, China; ^2^State Key Laboratory of Ecological Applied Technology in Forest Area of South China, Changsha, Hunan 410004, China

## Abstract

*Eucommia ulmoides *Oliv. (*E. ulmoides *Oliv.) and moso bamboo (*Phyllostachys pubescens*) leaves are used as folk medicines in central-western China to treat diabetes. To investigate the hypoglycemic activity of the effervescent granules prepared using* E. ulmoides *Oliv. and moso bamboo leaves (EBEG) in HepG2 cells, EBEG were prepared with 5% of each of polysaccharides and chlorogenic acids from moso bamboo and* E. ulmoides *Oliv. leaves, respectively. HepG2 cells cultured in a high-glucose medium were classified into different groups. The results displayed EBEG-treated cells showed better glucose utilization than the negative controls; thus, the hypoglycemic effect of EBEG was much greater than that of granules prepared using either component alone, thereby indicating that this effect was due to a synergistic action of the components. Further, glucose consumption levels in the cells treated with EBEG (156.35% at 200 *μ*g/mL) and the positive controls (metformin, 162.29%; insulin, 161.52%) were similar. Thus, EBEG exhibited good potential for use as a natural antidiabetic agent. The hypoglycemic effect of EBEG could be due to the synergistic action of polysaccharides from the moso bamboo leaves and chlorogenic acids from* E. ulmoides *Oliv. leaves via the inhibition of alpha-glucosidase and glucose-6-phosphate displacement enzyme.

## 1. Introduction

Diabetes mellitus (DM) is a metabolic disorder characterized by hyperglycemia and impaired metabolism of carbohydrates, proteins, and fats [1]. The World Health Organization lists DM as the third major cause of mortality, after cardiovascular disease and cancer. DM involves many complex pathological changes and the currently available chemical preparations used as antidiabetic agents have some limitations and are associated with several adverse effects, some being severe [2]. This state of affairs has prompted an increasing number of studies seeking to identify newer, safe, and effective hypoglycemic drugs, especially those sourced from natural substances such as plants [3].

*Eucommia ulmoides* Oliv. is a traditional Chinese medicinal herb. The barks of the plant are extensively studied and used, while its leaves are used in tea and as granules in food preparation.* E. ulmoides* Oliv. leaves have been reported to have hypolipidemic [4], antibacterial [5], hepatoprotective [6], and antihypertensive [7] activities, apart from other biological properties [8, 9]. Some of the important bioactive contents of* E. ulmoides* Oliv. leaves are chlorogenic acids, which are present at a concentration of up to 5% [10]. Studies have shown that chlorogenic acids have antidiabetic activity [11].

Members of the Bambusoideae subfamily are significant economic crops in the forestry field, and they are used as ingredients in food preparations and in medicinal formulations, besides other purposes [12]. Moso bamboo leaves have been reported to contain several compounds such as polysaccharides, phenolic acids, and flavones [13]. Polysaccharides from various plant sources have been shown to exhibit a range of pharmacological activities, including antitumor [14, 15], hypoglycemic [16, 17], immunomodulatory [18, 19], antiviral [20, 21], and other [22] activities. In fact, recent studies have shown that polysaccharides sourced from moso bamboo leaves have antidiabetic properties [23].

Thus, although the components of* E. ulmoides *Oliv. and those of moso bamboo leaves have been individually shown to have antidiabetic properties, the characteristics of a combination of these components remain to be examined. This study was aimed at examining the antidiabetic activity of effervescent granules prepared using polysaccharides from moso bamboo leaves and chlorogenic acids from* E. ulmoides *Oliv. leaves in cells of the human liver cancer cell line (HepG2 cells) by an* in vitro* glucose assay kit. Transfer of HepG2 cells cultured in low-glucose media to high-glucose media rapidly induces the expression of the gluconeogenic enzyme; therefore, we used* in vitro* cellular model HepG2 cells to analyze the hypoglycemic effects [24] of EBEG.

## 2. Materials and Methods

### 2.1. Source Plant Materials

Moso bamboo leaf extract, containing more than 40% polysaccharides and fillers, was purchased from Golden-Basin Biotechnology Co. Ltd. (Guangdong, China), while* E. ulmoides *Oliv. leaf extract containing 5% chlorogenic acids and fillers was purchased from Yuan-Hang Biotech Co. Ltd. (Hunan, China).

### 2.2. Preparation of Effervescent Granules

**Figure 1:**
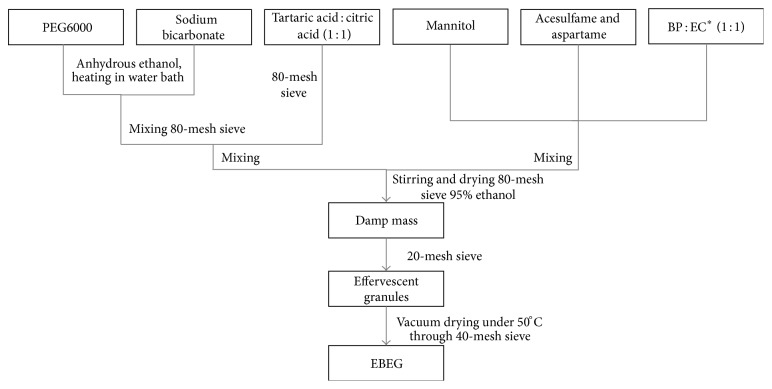
Flow chart depicting the preparation of EBEG (effervescent granules prepared using* E. ulmoides* Oliv. and moso bamboo leaves). ^*∗*^BP: polysaccharides from moso bamboo leaves; EC: chlorogenic acids from* E. ulmoides *Oliv. leaves.

A schematic representation of the method of preparing EBEG has been provided in Figure 1. In the process, sodium bicarbonate is added in a solution of PEG6000 in anhydrous ethanol, with heating in water bath. This combination needs to be dried, poured through 80-mesh sieve, and added to the blended tartaric acid and citric acid. Thereafter, the following order is mixed with a combination of flavoring agents (mannitol) and sweeteners (acesulfame and aspartame). The powder is granulated by being moistened with about 95% ethanol and sieved through 80-mesh sieve. When damp, this product is sieved through a 20-mesh sieve and dried under 50°C. The dried granules are again screened through a 40-mesh sieve to break up any agglomeration and packaged in moisture tight containers.

Effervescent granules containing only polysaccharides from moso bamboo leaves (BPEG) or chlorogenic acids from* E. ulmoides *Oliv. Leaves (ECEG) were also prepared for use as negative controls. The procedure for the preparation of BPEG and ECEG was similar to the one used for the preparation of EBEG. Effervescent granules (EG) were also prepared without the addition of any of the abovementioned components and only the vehicle.

The content of chlorogenic acid in the effervescent granules was analyzed using high performance liquid chromatography (HPLC) with conditions shown in Table 1 [25]. The obtained average content of chlorogenic acid in the effervescent granules was 2.4 mg/g.

The content of polysaccharides in the effervescent granules was analyzed using phenol-vitriolic colorimetry [25]. Fresh phenol and concentrated sulfuric acid were added to a series of diluted concentrations of anhydrous glucose solution and kept in the boiling water bath for 15 min. The standard curve was established by monitoring the dependence of 490 nm absorbance on the anhydrous glucose concentration. According to the standard curve, the average content of polysaccharides was 24.18 mg/g.

### 2.3. Cell Culture and Cytotoxicity Studies

Human HepG2 cells were provided by the National Engineering Laboratory of Rice and By-Products Processing. The cells were maintained in Dulbecco's modified Eagle's medium (DMEM; HyClone, Logan, USA) supplemented with 10% fetal bovine serum (FBS) (Fame Biotechnology, China) and the antibiotics penicillin and streptomycin, at 37°C in an atmosphere of 5% CO_2_. To determine the effect of EBEG, the cells were first incubated for 24 h in serum-free RPMI-1640 (low-glucose medium). Thereafter, the cells were incubated in DMEM (high-glucose concentration of 22.2 mmol/L) in the presence of EG, EBEG, BPEG, ECEG, metformin, or insulin. In addition, one group of cells was left untreated to serve as the untreated control.

### 2.4. Glucose Utilization

**Figure 2:**
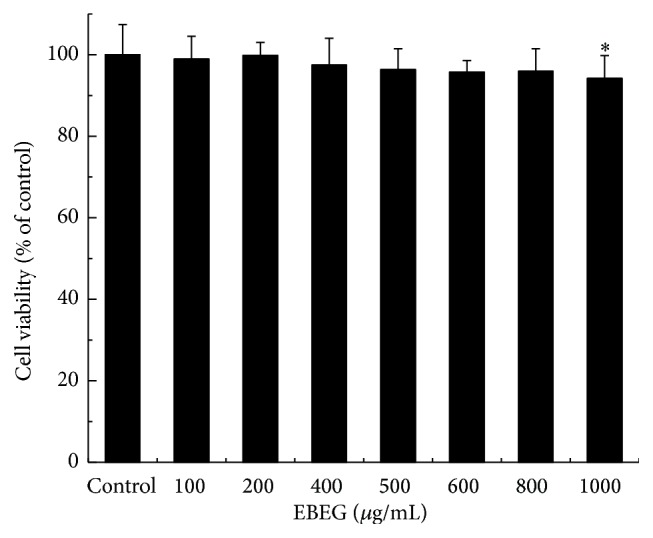
Effect of EBEG (effervescent granules prepared using* E. ulmoides* Oliv. and moso bamboo) leaves exposure on HepG2 cell viability. HepG2 cells were treated with various concentrations of EBEG, and the cytotoxicity level was determined by MTS analysis. Data points represent mean ± SD; *N* = 6 wells in a 96-well plate. Compared with the control group, ^*∗*^ *P* < 0.05.

Cell toxicity studies were first performed to determine the toxicity threshold of EBEG concentration by using 3-(4,5-dimethylthiazol-2-yl)-5(3-carboxymethonyphenol)-2-(4-sulfophenyl)-2H-tetrazolium (MTS) method, as shown in Figure 2. Since EBEG displayed toxicity at concentrations above 800 *μ*g/mL, concentrations below 500 *μ*g/mL were used in all subsequent experiments.

The glucose utilization assay was conducted at 37°C in 5% CO_2_. HepG2 cells were seeded into 96-well culture plates at a density of 4000 to 5000 cells (100 *μ*L) per well and cultured for 4 h. Thereafter, the cells were starved until the day of the glucose utilization experiment, to allow for the depletion of glucose from the medium.

Next, 100 *μ*L of the incubation medium (DMEM + 10% FBS) containing the control, positive control, negative controls, or sample at the appropriate concentrations was added to the wells, with 6 wells for each group of cells. The wells for the untreated controls contained only the incubation medium. The wells for the positive controls were treated with 60 *μ*g/mL metformin or 20 unit/mL insulin. The cells in the negative control and experimental sample were treated with BPEG, ECEG, or EG and EBEG, respectively, at concentrations of 200 *μ*g/mL, 400 *μ*g/mL, or 500 *μ*g/mL. The concentrations were determined according to the results of the preliminary cytotoxicity test. Solutions of EBEG, ECEG, BPEG, and EG were prepared by reconstituting them in sterilized water. The cells were incubated under these conditions for 24 h at 37°C. 1 *μ*L of the solution was removed from each well and placed into a new 96-well plate into which 100 *μ*L of the growth medium provided with the glucose assay kit (Huili Biotech, China) was added. The plates were incubated for a further 15 min at 37°C, and the absorbance was measured at 492 nm by using the Multiscan microtiter plate reader (Prolong, Beijing, China). To assess the glucose utilization, glucose consumption (GC) by the cells was calculated as the amount of glucose left in the medium after incubation subtracted from the initial amount. The glucose uptake was calculated using the following formula:

(1) GCmmol/L=22.2−ODSampleODStandard×CStandardGlucose  Uptake%=GCSampleGCControl×100%.

### 2.5. Statistical Analysis

The results of the glucose utilization with the different controls and samples were compared by Student's* t*-test using IBM Corp., released 2010, IBM SPSS Statistics for Windows, Version 19.0. (Armonk, NY: IBM Corp.) for statistical analysis and represented as mean ± SD. *P* value of <0.05 was considered significant, and *P* of <0.01 was considered highly significant.

## 3. Results

**Figure 3:**
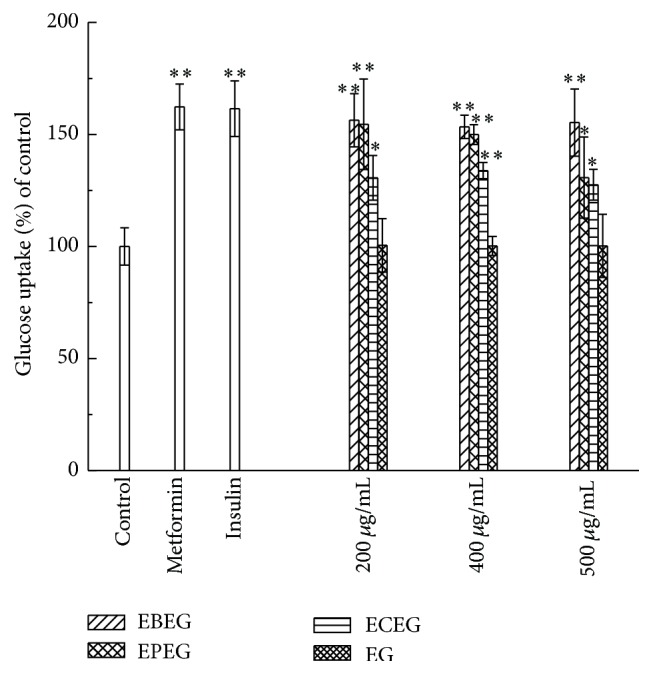
Effect of exposure to various concentrations EBEG (effervescent granules prepared using* E. ulmoides *Oliv. and moso bamboo leaves), BPEG (effervescent granules containing only polysaccharides from moso bamboo leaves), ECEG (Effervescent granules containing only chlorogenic acids from* E. ulmoides *Oliv. leaves), EG (effervescent granules containing only the vehicle), metformin, and insulin on glucose uptake in HepG2 cells in comparison with untreated control cells. Cells were exposed to EBEG, BPEG, ECEG, and EG at concentrations of 200 *μ*g/mL, 400 *μ*g/mL, and 500 *μ*g/mL; 60 *μ*g/mL of metformin; or 20 unit/mL insulin for 24 h; and glucose uptake was measured in the presence of glucose oxidase. Data points represent mean ± SD; *N* = 6 wells in a 96-well plate. Compared with the control group, ^*∗*^ *P* < 0.05 and ^*∗∗*^ *P* < 0.01.

The results of the present study are summarized in Figure 3. The response of the untreated control cells was taken as 100%. The response of the cells treated with vehicle for EG alone was similar to that of the untreated controls, indicating that the process of preparing the granules had no effect on the glucose consumption of the cells. Further, the glucose uptake of EBEG was the higher than that of BPEG and ECEG at all the tested concentrations, indicating that its hypoglycemic activity was much greater than those of BPEG and ECEG alone. This, in turn, implies that the individual components of EBEG acted synergistically to produce a greater hypoglycemic effect. Further, at a concentration of 200 *μ*g/mL, EBEG exhibited a glucose uptake of 156.35%, which was similar to that achieved with metformin treatment (162.29%) and insulin treatment (161.52%) under the same experimental conditions. This implies that the hypoglycemic effect of EBEG was similar to that of known antidiabetic agents.

## 4. Discussion

In this study, we prepared EBEG by combining polysaccharides extracted from moso bamboo leaves and chlorogenic acids extracted from* E. ulmoides *Oliv. leaves. We compared the antidiabetic effects of EBEG with those of BPEG and ECEG individually and found that the hypoglycemic activity of the EBEG was much greater than those of the individual components. Furthermore, the vehicular components of EG exerted no influence on the antidiabetic activity of both chlorogenic acids and polysaccharides.

As one of the most important organs for glucose metabolism, liver plays a significant role in maintaining glucose homeostasis, especially in the gluconeogenesis, glycogen synthesis, and glucose uptake, utilization, and release. When the blood glucose concentration is low, glycogen is mobilized and converted to glucose by gluconeogenesis. When glucose supply exceeds the body need, however, the glycogen synthesis is promoted and the glycogenolysis is inhibited to maintain normal levels of glucose in the blood [26]. Abnormal glucose metabolism is accompanied by insulin resistance, which is the typical pathophysiological phenomenon for type 2 diabetes [27, 28].

HepG2 is a human liver carcinoma cell line with similar phenotype and biological feature of normal liver cells, which has been widely used in the diabetes research and treatment. HepG2 takes up the glucose to synthesize glycogen and maintain normal levels of glucose. The result in this paper revealed that* E. ulmoides *Oliv. and moso bamboo leaves (EBEG) significantly enhanced the uptake of glucose in HepG2 and decreased the blood glucose concentration, which is the hypoglycemic mechanism for EBEG.

In truth, HepG2 is just a cell line which cannot exactly simulate the complex and multienzyme regulated glucose metabolism* in vivo*. Therefore, we tried to figure out the underlying mechanism consulting the references and explored the hypoglycemic activity of EBEG in depth.

Glucose-6-phosphatase plays a major role in the regulation of blood glucose homeostasis by facilitating the formation of endogenous glucose during gluconeogenesis and glycogenolysis. Recent studies have shown that chlorogenic acids are specific inhibitors of glucose-6-phosphate translocase—a component of this enzyme system. In fact, both* in vivo* and* in vitro* studies have proven that chlorogenic acids have antidiabetic properties. Glucose-6-phosphatase translocase inhibitors have been shown to mitigate the inappropriately high levels of hepatic glucose production in patients with non-insulin-dependent diabetes [29]. Another similar study [30] showed that the acute inhibition of glucose-6-phosphatase activity by the chlorogenic acid derivative S4048 had a two-pronged effect on hepatic carbohydrate fluxes in isolated rat hepatocytes and in rats: newly synthesized glucose-6-phosphatase was redirected from glucose production to glycogen synthesis without compromising the gluconeogenic flux to glucose-6-phosphatase and a cellular response was triggered for the maintenance of the cellular levels of glucose-6-phosphatase. Similarly, chlorogenic acids extracted from honeysuckle have been shown to induce hypoglycemia in mice [11], probably via the inhibition of glucose-6-phosphate displacement enzyme and glucose absorption. This result is consistent with our finding that the effect of EBEG on the glucose consumption of HepG2 cells may be mediated by the inhibition of the glucose-6-phosphate displacement enzyme.

Polysaccharides in food are known to improve health and prevent diseases such as diabetes. Several studies have shown that polysaccharides exert hypoglycemic effects, possibly via the inhibition of the alpha-glucosidase activity [31]. Polysaccharides present in green tea have been shown to have antidiabetic effects [32], while those extracted from* Taxus chinensis *var.* mairei* are reported to increase the glucose consumption of HepG2 cells [33]; the mechanism of action is believed to be inhibition of alpha-glucosidase activity in both these cases. Likewise, polysaccharides extracted from moso bamboo leaves (PMBL) have been shown to have a good hypoglycemic effect on alloxan diabetic mice, thereby suggesting that the hypoglycemic effect of PMBL was associated with not only the inhibition of alpha-glucosidase but also the enhancement of the effects of liver glycogen and muscle glycogen [23]. Consistent with these findings, our results showed that BPEG exhibited hypoglycemic activity in HepG2 cells; further investigations that include an assay for measuring alpha-glucosidase inhibition* in vitro* would help confirm the mechanism of action underlying this effect.

The polysaccharide of bamboo usually refers to the compound consisting of more than 10 monosaccharides but excludes starch, cellulose, and pectin. Commercial polysaccharide of bamboo is usually produced through specific extraction technique with trace ingredients addition.* E. ulmoides *Oliv. leaves contain chlorogenic acid, flavone, iridoid, and terpene compounds. In the commercial* E. ulmoides *Oliv. leaves extract, however, only one specific compound is the main component. In this paper, the main component of* E. ulmoides *Oliv. leaves extract was chlorogenic acid, so that other ingredients were in low amount.

In traditional Chinese medicine, various medicinal substances are used in conjunction to prepare a formula or prescription that can produce the desired therapeutic effect while reducing the toxic or adverse effects of the individual components; this concept in traditional Chinese medicine is called mutual reinforcement. In EBEG, both chlorogenic acids from* E. ulmoides *Oliv. leaves and polysaccharides from moso bamboo leaves exerted a synergistic action to produce a hypoglycemic effect that was stronger than those of the individual components and similar to the effect of known hypoglycemic agents. Together, the individual components enhanced the inhibition of both alpha-glucosidase and glucose-6-phosphate displacement enzyme, thereby reducing the rate of glycogen metabolism and also promoting the synthesis of hepatic glycogen. The findings of this study enrich our understanding of the role of* E. ulmoides *Oliv. and moso bamboo in treating diabetes mellitus. It is possible that other traditional Chinese medicinal herbs might have similar effects, thereby highlighting the need for further investigation. Nevertheless, our findings need to be confirmed by further investigations at the cellular and molecular levels as well as studies on structure-activity relationship to clarify the mechanism of action in detail.

The results of this study suggest that EBEG has potential for use as a natural health product to combat diabetes and that its main components act synergistically to produce a strong hypoglycemic effect. Further animal and clinical studies are warranted to determine whether EBEG can be recommended on a regular basis for the management of blood glucose levels in diabetic patients.

## Figures and Tables

**Table 1:** Parameter details.

Parameter	Detail
ODS chromatographic column	250 mm × 4.6 mm, 5 *μ*m
Mobile phase	Acetonitrile: 1% acetic anhydride (1 : 9)
Flow rate	1.0 mL/min
Sample volume	10 *μ*L
Wavelength	327 nm
